# An inverse association between West Nile virus serostatus and avian malaria infection status

**DOI:** 10.1186/1756-3305-7-415

**Published:** 2014-09-01

**Authors:** Matthew CI Medeiros, Tavis K Anderson, Jenni M Higashiguchi, Uriel D Kitron, Edward D Walker, Jeffrey D Brawn, Bethany L Krebs, Marilyn O Ruiz, Tony L Goldberg, Robert E Ricklefs, Gabriel L Hamer

**Affiliations:** Department of Biology, University of Missouri-St. Louis, One University Boulevard, St. Louis, MO 63121 USA; Department of Biology, Georgia Southern University, P.O. Box 8042–1, Statesboro, 30460 Georgia; Department of Environmental Studies, Emory University, 400 Dowman Drive, Atlanta, GA 30322 USA; Department of Microbiology and Molecular Genetics, 2215 Biomedical Physical Sciences East, Lansing, MI 48824-4320 USA; Department of Natural Resources and Environmental Sciences, University of Illinois, 1102 South Goodwin Ave., Urbana, IL 61801 USA; Department of Pathobiology, University of Illinois, 2001 S. Lincoln, Urbana, IL 61801 USA; Department of Pathobiological Sciences, University of Wisconsin, 2015 Linden Dr., Madison, WI 53706 USA; Department of Entomology, Texas A&M University, 2475 TAMU, College Station, TX 77843-2475 USA

**Keywords:** Parasite-parasite interactions, Coinfection, Concurrent infection, Concomitant infection, West Nile virus, Haemosporida, Avian malaria

## Abstract

**Background:**

Various ecological and physiological mechanisms might influence the probability that two or more pathogens may simultaneously or sequentially infect a host individual. Concurrent infections can have important consequences for host condition and fitness, including elevated mortality risks. In addition, interactions between coinfecting pathogens may have important implications for transmission dynamics.

**Methods:**

Here, we explore patterns of association between two common avian pathogens (West Nile virus and avian malaria parasites) among a suburban bird community in Chicago, IL, USA that share mosquito vectors. We surveyed 1714 individual birds across 13 species for both pathogens through established molecular protocols.

**Results:**

Field investigations of haemosporidian and West Nile virus (WNV) infections among sampled birds yielded an inverse association between WNV serostatus and *Plasmodium* infection status. This relationship occurred in adult birds but not in juveniles. There was no evidence for a relationship between *Haemoproteus* infection and WNV serostatus. We detected similar prevalence of *Plasmodium* among birds captured with active WNV infections and spatiotemporally paired WNV-naïve individuals of the same species, demonstrating that the two pathogens can co-infect hosts.

**Conclusions:**

Mechanisms explaining the negative association between WNV serostatus and *Plasmodium* infection status remain unclear and must be resolved through experimental infection procedures. However, our results highlight potential interactions between two common avian pathogens that may influence their transmission among hosts. This is especially relevant considering that West Nile virus is a common zoonotic pathogen with public health implications. Moreover, both pathogens are instructive models in infectious disease ecology, and infection with either has fitness consequences for their avian hosts.

**Electronic supplementary material:**

The online version of this article (doi:10.1186/1756-3305-7-415) contains supplementary material, which is available to authorized users.

## Background

Numerous pathogens co-circulate within host populations, and various mechanisms influence the probability that these pathogens may cause concurrent or consecutive infections within a host individual [1–4]. For instance, pathogens with similar ecological tolerances and vectors might be more likely to co-occur within a host [5], while differences may result in non-overlapping distributions across hosts over space and time. Infection can influence the susceptibility of a host to another pathogen [2, 4], and this interaction can alter pathogen transmission dynamics at the population level [1]. Negative associations between pathogens occur when an established pathogen lowers the infection success of another pathogen through immune-mediated mechanisms [3, 4], resource competition, or host mortality [4]. Conversely, established parasites can increase the probability of infection of another pathogen through facilitation [1, 2]. Concurrent infections can elevate host morbidity and mortality rates [6], although this effect often depends on the particular pathogens involved [7, 8]. Thus, pathogen-pathogen interactions can greatly influence the course of infection within the host and the distribution of pathogens among host individuals at the population level.

Avian Haemosporida are an instructive model system for disease ecology, including the consequences of concurrent infections. Mixed avian haemosporidian infections can be common within individuals in some host populations [9]. Both field- and laboratory-based studies have demonstrated that co-infection with different haemosporidian parasites can depress host fitness more than single infections [10, 11]. However, few studies have explored the interactions of avian Haemosporida with other pathogens (in contrast to mammalian malaria [7, 12], however, see Atkinson *et al*. [5], Barnett [13]). This absence of knowledge is particularly concerning because birds are primary reservoirs for many zoonotic pathogens, including arthropod-borne encephalitis viruses whose course of infection may be influenced by concurrent protozoan infections [12]. Thus, understanding interactions between Haemosporida and other pathogens could have important implications for disease surveillance and animal and human health.

Here, we explore patterns of association between avian Haemosporida and West Nile virus (WNV), an important zoonotic pathogen. We consider the genera *Plasmodium* and *Haemoproteus*, which are diverse and abundant Haemosporida that infect a range of avian host species [14]. The two genera differ in their lifecycles and transmission dynamics [14, 15]. Most notably, *Plasmodium* replicates asexually within erythrocytes leading to the rupture of blood cells, and is vectored by mosquitoes (Culicidae). In *Haemoproteus* infections, asexual reproduction is limited to the viscera and vascular endothelium, and the parasites are vectored by *Culicoides* biting midges (Ceratopogonidae). Haemosporida parasites of both genera are often pathogenic [16–18], but virulence varies with pathogen lineage and host species [18, 19]. Infections can maintain throughout the life of the host, but may disappear from the bloodstream and relapse later [14]. Vernal recrudescence, a phenomenon in which dormant infections acquired during previous transmission seasons relapse into the bloodstream in the spring, is common in temperate latitudes [14]. WNV was introduced to North America in 1999 and spread across most of the continent within 5 years [20, 21]. In 2012, one of the largest WNV epidemics to date occurred in North America (CDC). WNV is maintained in a transmission cycle generally between mosquitoes and birds, but is occasionally transmitted to other hosts, including humans [21]. Symptoms of WNV infection in humans may be mild to severe, occasionally including neurologic impairment or death [22]. WNV infection also has severe fitness consequences for some avian host species [23], and its introduction coincided with population declines in some North American bird species [24]. Associations between WNV and trypanosomes [25] and *Culex* flavivirus [26] have previously been documented in mosquitoes. However, interactions of WNV with Haemosporida within birds have received comparatively little attention.

The similar ecology of avian Haemosporida and WNV suggests that interactions between these pathogens might occur in North America. Both WNV and avian Haemosporida are common in American robins (*Turdus migratorius*), northern cardinals (*Cardinalis cardinalis*), and house sparrows (*Passer domesticus*) [27]. Individuals of these species also host WNV [28, 29] and appear to be the vertebrate drivers of local WNV transmission dynamics in the eastern Unites States [30, 31]. In addition, WNV and avian *Plasmodium* share the same vectors (*Culex pipiens* and *Cx. restuans*) in eastern North America [14, 27, 32–34], suggesting similar host encounter rates between the pathogens. In this study, we explore the potential interaction between WNV and Haemosporida near Chicago, IL, USA, and show that WNV seropositive birds have a lower probability of haemosporidian infection.

## Methods

### Sampling and pathogen testing

The study was conducted at 17 sites in suburban Chicago, IL, USA [35]. Birds were captured in mist-nets from May-October during 2006–2007 and screened for avian Haemosporida [27]. A blood sample was taken from the jugular vein and centrifuged to separate serum from blood cells. Packed blood cells were preserved in Longmire’s lysis buffer. Samples were digested with Proteinase K overnight at 60°C. DNA was extracted via protein precipitation with 5 M ammonium acetate, and purified with a standard alcohol precipitation. DNA samples were screened for haemosporidian parasites by polymerase chain reaction (PCR) targeting a segment of the mitochondrial 16S rRNA gene [36]. Samples that tested positive by this method were then subjected to a nested PCR that targeted a 552-base pair fragment of the haemosporidian cytochrome *b* gene [37, 38]. We generally obtained sequences from ~85% of samples that screened positive for a haemosporidian infection with the 16S rRNA primers.

Avian haemosporidian taxonomy is unresolved at the species level, and currently relies on cytochrome *b* sequences to identify parasite taxa [39–41]. We therefore separated evolutionary lineages of *Plasmodium* and *Haemoproteus* following Ricklefs *et al.* [37]. Generally, haemosporidian taxa were categorized as sets of closely related (<1% sequence divergence) monophyletic parasite cytochrome *b* haplotypes recovered from the same set of host species. Two lineages presented here were identical to previously named morphospecies (*Plasmodium cathemerium* and *Plasmodium elongatum,* Genbank accession no. AY377128 and AY733088, respectively).

Avian serum was used to test for the presence of WNV antibodies using inhibition ELISA, and to screen individuals for circulating WNV with a quantitative reverse transcriptase-PCR (methodology for the ELISA and quantitative reverse transcriptase-PCR summarized in Hamer *et al.* [42]). Among birds screened for malaria, ~7% were seropositive for WNV antibodies. 5728 birds (including birds screened for haemosporian parasites here) were screened for WNV [35, 43] across an extended WNV study from 2005–2011 in the study site. Only 27 (0.5%) individuals were positive for the virus by RT-PCR [43]. To compare concurrent infection rates of WNV and Haemosporida, blood samples from 26 of these birds were screened for haemosporidian infections. In addition, we screened 26 spatiotemporally paired WNV-negative samples of the same species and age. Each WNV positive and negative pair was sampled from the same site, generally on the same day.

### Haemosporidian phylogeny

We aligned cytochrome *b* sequences (512 bp and subsequently inferred phylogenetic relationships among haemosporidian parasites using maximum-likelihood methods, invoking a general time-reversible (GTR) model of nucleotide substitution with Γ-distributed rate variation among sites in MEGA5 [44]. Statistical support was estimated for individual nodes by bootstrap analysis (1000 replicates), and the best-scoring tree was mid-point rooted for clarity. The resulting tree (Additional file 1: Figure S1) was used primarily to assign lineages to genera. Sequences of all unique cytochrome *b* lineages are deposited in GenBank (accession no. KC789821–KC789828, KM280598– KM280635.

### Statistical analyses

We used a series of generalized linear mixed models (GLMM) with a binomial error distribution to test for an association between WNV serostatus and Haemosporida infection status for birds sampled between 2006–2007. All GLMMs were performed in the lme4 package in R. Our data were heterogeneous and unbalanced with respect to other variables that potentially influence haemosporidian infection across WNV-seropositive and naïve individuals. Therefore, we included species as a random factor in all models tested. Moreover, year of sampling (two levels: 2006 and 2007), month of sampling (four levels: May/June, July, August, September/October), age class at sampling (two levels: hatch-year juvenile [HY] or after hatch-year adult [AHY]), WNV serostatus (presence or absence of WNV antibodies), and an age*WNV serostatus interaction, were included as covariates in a full model.

We used AICc multimodel inference to select among a set of candidate models (SOM-Section 2) that included all combinations of the five fixed effect variables. We estimated the natural average of the estimate and associated unconditional 95% confidence intervals [45] with the R package “AICcmodavg”. WNV serostatus model estimates (*β*_*WNV*_) represent the change in the log-odds of Haemosporida infection for WNV seropositive relative to WNV seronegative individuals. Within the text, values of *β*_*WNV*_ are presented as the natural average of the model coefficients ± 1.96 * unconditional (model-averaged) standard error (SE). When calculating the model-averaged *β*_*WNV*_, we excluded models with the age*WNV interaction term. We performed the basic modeling approach detailed above for five separate analyses in which the dependent variable of infection status was defined differently: 1) total Haemosporida; 2) total *Plasmodium*; 3) total *Haemoproteus*; 4) *Plasmodium cathemerium*; and 5) *Plasmodium elongatum*. We performed the basic modeling approach detailed above for data on 1714 individuals of 13 well-sampled species (N > 10) that had both haemosporidian infections and WNV seropositive individuals. We excluded records of other species that did not fit the criteria above, or those with missing data (ie. unknown age, WNV serological status, etc.). All species along with their sample sizes across age class, *Plasmodium* prevalence, *Haemoproteus* prevalence, and WNV seroprevalence are listed in Additional file 1: Table S1.

### Ethical approval

Fieldwork was authorized by the appropriate permits including a Federal Bird Banding Permit no. 06507, animal-use approvals from the University of Illinois Animal Use Protocol no. 03034, and Institutional Animal Care and Use Committee at Michigan State University, Animal Use Form no. 12/03-152-00.

## Results

### WNV serostatus and haemosporida infection

Among the community-level dataset, the prevalence of *Plasmodium* and *Haemoproteus* parasites was 0.26 and 0.08 respectively. Seroprevalence of WNV antibodies was 0.07.

The best-fit model explaining total Haemosporida infection status included month of capture, year of capture, age, WNV serostatus, and age*WNV serostatus interaction (*w*_*i*_ =0.97), and was differentiated from other models (Table 1). Thus, we split the dataset across age class to investigate the interaction. For adults, WNV serostatus was an important predictor of Haemosporida infection (Table 2). The best-fit model included year and WNV serostatus (*w*_*i*_ = 0.69) and was differentiated from a model that only included year as a fixed effect (ΔAICc = 5.5, *w*_*i*_ = 0.04). The model-averaged WNV serostatus effect (*β*_*WNV*_= − 0.78 ± 0.59) indicated that the presence of WNV antibodies reduced the odds of a concurrent haemosporidian infection by a factor of 2.2 for adult birds. Among juvenile birds, the best-fit model included month and year of capture (*w*_*i*_ = 0.73), and was differentiated from a model that contained month, year, and WNV serostatus (ΔAICc = 2.0, *w*_*i*_ = 0.27). The confidence limits of the WNV serostatus effect (*β*_*WNV*_= − 0.06 ± 0.94) for juvenile hosts included zero, providing low support for an association between WNV serostatus and Haemosporida infection among hatch-year birds.

**Table 1:** A summary AICc table for generalized linear mixed models that considered both host ages simultaneously and an age*WNV serostatus interaction effect

	K	AICc	ΔAICc	***w***_***i***_AICc
**a) Total Haemosporida**				
mon+yr+age+**wnv**+wnv*age	9	2056.5	0.0	0.97
mon+yr+age+**wnv**	8	2064.0	7.5	0.02
**b) Total** ***Plasmodium***				
mon+yr+age+**wnv**+wnv*age	9	1704.7	0.0	0.99
mon+yr+age+**wnv**	8	1713.9	9.1	0.01
**c) Total** ***Haemoproteus***				
mon	5	742.3	0.0	0.32
mon+yr	6	744.1	1.8	0.13
mon+age	6	744.2	1.9	0.12
mon+**wnv**	6	744.3	2.0	0.12
**d)** ***P. cathemerium***				
mon+yr+age+**wnv**	8	823.4	0.0	0.59
mon+yr+age+**wnv**+wnv*age	9	825.2	1.8	0.24
**e)** ***P. elongatum***				
yr+age+**wnv**	5	586.23	0.0	0.40
yr+age+**wnv**+wnv*age	6	587.0	0.8	0.27
yr+age	4	587.6	1.4	0.20

Models with ΔAICc < 3.0 are shown. However, when only one model had a ΔAICc < 3.0, the next best model was listed for comparison. Abbreviations are as follows: mon = month of capture, yr = year of capture, age = age class, wnv = WNV serostatus (seropositive, seronegative). Species was a random effect in all models tested.

**Table 2:** **A summary AICc table for generalized linear mixed models performed on adults and juveniles separately**

	K	AICc	ΔAICc	***w***_***i***_AICc
**a) Total Haemosporida-adults**				
yr+**wnv**	4	1138.9	0.0	0.69
mon+yr+**wnv**	7	1140.9	2.1	0.24
**b) Total Haemosporida-juveniles**				
mon+yr	6	862.7	0.0	0.73
mon+yr+**wnv**	7	864.7	2.0	0.27
**c) Total** ***Plasmodium*** **-adults**				
yr+**wnv**	4	871.4	0.0	0.64
mon+yr+**wnv**	7	872.6	1.2	0.35
**d) Total** ***Plasmodium*** **-juveniles**				
mon+yr	6	785.5	0.0	0.72
mon+yr+**wnv**	7	787.4	1.9	0.28

Only models with ΔAICc < 3.0 are shown. *Abbreviations* are as follows: mon = month of capture, yr = year of capture, wnv = WNV serostatus (seropositive, seronegative). Species was a random effect in all models tested.

**Figure 1:**
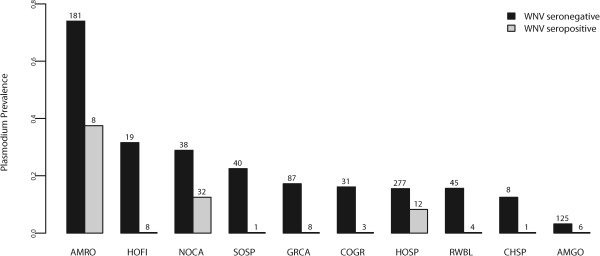
***Plasmodium*** **prevalence of WNV-seropositive and WNV-seronegative adult birds across host species.** The numbers above bars represent sample sizes. European starlings (*Sturnus vulgaris*), mourning doves (*Zenaida macroura*), and brown-headed cowbirds (*Molothrus ater*) were not included because of a total lack of *Plasmodium* or WNV infections among adult birds. Labels correspond to 4-letter abbreviated American Ornithologists’ Union alpha codes. AMRO = American robin (*Turdus migratorius*), HOFI = house finch (*Carpodacus mexicanus*), NOCA = northern cardinal (*Cardinalis cardinalis*), SOSP = song sparrow (*Melospiza melodia*), GRCA = gray catbird (*Dumetella carolinensis*), COGR = common grackle (*Quiscalus quiscula*), HOSP = house sparrow (*Passer domesticus*), RWBL = red-winged blackbird (*Agelaius phoeniceus*), CHSP = chipping sparrow (*Spizella passerina*), AMGO = American goldfinch (*Carduelis tristis*).

The best-fit model explaining total *Plasmodium* infection status included month of capture, year of capture, age, WNV serostatus, and age*WNV serostatus interaction (*w*_*i*_ =0.99), and was differentiated from other models (Table 1). Splitting the dataset across age classes, the best-fit model for adult birds included year and WNV serostatus (*w*_*i*_ =0.64, Table 2), but was indistinguishable from a model that included month, year, and WNV serostatus (ΔAICc = 1.2, *w*_*i*_ = 0.35). The model-averaged WNV serostatus effect (*β*_*WNV*_= − 1.34 ± 0.84) indicated that the presence of WNV antibodies reduced the odds of a concurrent *Plasmodium* infection by a factor of 3.8 for adult birds (Figure 1). For juvenile birds, the best-fit model included month and year of capture (*w*_*i*_ = 0.72, Table 2), but was indistinguishable from a model that included those fixed effects and WNV serostatus (ΔAICc = 1.9, *w*_*i*_ = 0.28). The confidence limits of the WNV serostatus effect (*β*_*WNV*_= âˆ’0.18 ± 0.94) for juvenile hosts included zero, providing low support for an association between WNV serostatus and *Plasmodium* infection among hatch-year birds.

Asynchronous infection dynamics between WNV and *Plasmodium* in the study site could explain the inverse relationship between WNV serostatus and the probability of a *Plasmodium* infection among adult birds. Generalized linear mixed models with binomial error distributions and species as a random effect revealed that the probability of having WNV antibodies or a *Plasmodium* infection differed across months. WNV seroprevalence in adult birds increased across the transmission season (likelihood ratio test of nested models, *χ*^2^=8.6, df = 3, p < 0.05). WNV seroprevalence was lowest in May-June, moderate in July and August, and highest in September-October. In contrast, the probability of *Plasmodium* infection was consistent across the season (likelihood ratio test of nested models, *χ*^2^=2.6, df = 3, p > 0.4). The different temporal patterns of WNV seropositve and *Plasmodium* infected hosts across the transmission season did not solely drive the negative association between these variables. WNV serostatus remained an important negative predictor of *Plasmodium* infection status (Additional file 1: Table S11) among adult hosts sampled during the later portion of the transmission season (July through October) when there was more overlap between *Plasmodium*-infected and WNV-seropositive individuals. The model-averaged WNV serostatus effect (*β*_*WNV*_= − 1.71 ± 1.18) indicated that the presence of WNV antibodies reduced the odds of a *Plasmodium* infection by a factor of 5.5 for adult birds.

We found no support for an association between WNV serostatus and *Haemoproteus* infection (Table 1). The best-fit model included month (*w*_*i*_ = 0.32), however, three other models had ΔAICc < 3 (Table 1). The confidence limits of the WNV serostatus effect on *Haemoproteus* included zero (*β*_*WNV*_= 0.08 ± 0.67).

Results with two well-sampled *Plasmodium* lineages were broadly similar to those obtained with total *Plasmodium*. The best fit model for *Plasmodium cathemerium* included the fixed effects of month, year, age, and WNV serostatus (*w*_*i*_ = 0.59, Table 1), but was indistinguishable from a model that included those fixed effects and the age*WNV serostatus interaction (ΔAICc = 1.8, *w*_*i*_ = 0.24). However, the best-fit model was differentiated from a model that included only month, year, and age (ΔAICc = 4.0, *w*_*i*_ = 0.08). The model-averaged WNV serostatus effect (*β*_*WNV*_= − 1.22 ± 1.10) indicated that the presence of WNV antibodies reduced the odds of a concurrent *P. cathemerium* infection by a factor of 3.4 for adult birds. The best-fit model for *Plasmodium elongatum* included the fixed effects of year, age, and WNV serostatus (*w*_*i*_ = 0.40, Table 1), but was indistinguishable from a model that included only year and age (ΔAICc = 1.4 *w*_*i*_ = 0.20). The model-averaged WNV serostatus estimate (*β*_*WNV*_= − 0.89 ± 1.0) indicated that the presence of WNV antibodies reduced the odds of a concurrent *P. elongatum* infection by a factor of 2.4, although the confidence limits of the estimate included zero. Full AICc tables for all analyses are shown in the Additional file 1: Tables S2-11.

### Analysis of WNV-positive birds

Nine birds of 26 that were positive for WNV had Haemosporida infections, eight of which were *Plasmodium*. A similar number (8/26) of spatiotemporally paired individuals of the same species that did not test positive for WNV had haemosporidian infections (p = 1.0, fisher exact test). All of these were *Plasmodium* infections (Table 3). WNV-infected birds appear to be infected with a diversity of Haemosporida at a comparable rate relative to birds without WNV infections (Table 3).

**Table 3:** **Number of Haemosporida infections and total host individuals sampled among birds with active WNV infections and spatiotemporally paired individuals of the same age and host species that were naïve to WNV**

a) West Nile virus positive				
	Haemosporida infections	***Plasmodium***infections	Total sampled	Haemosporidian Lineages
American robin^1^	2	1	2	CHI04PL, CHI02PL, CHI19PA
Gray catbird	1	0	1	CHI01PA
House finch	0	0	3	
House sparrow	4	4	15	*P. cathemerium*, CHI05PL, *P. elongatum*
House wren	0	0	1	
Northern cardinal	2	2	2	*P. cathemerium*, *P. elongatum*
Red-winged blackbird	0	0	1	
Northern flicker	0	0	1	
Total	9	7	26	
**b) West Nile virus negative**				
American robin^1^	1	1	2	CHI04PL^1^, *P. elongatum*
Gray catbird	0	0	1	
House finch	1	1	3	*P.cathemerium*
House sparrow^2^	4	2	15	*P.cathemerium*, *P. elongatum*
house wren	0	0	1	
Northern cardinal	1	1	2	*P. elongatum*
Red-winged blackbird	1	1	1	*P.cathemerium*
Northern flicker	0	0	1	
Total	8	6	26	

Haemosporida lineages recovered from each host are listed.

^1^Two *Plasmodium* lineages were recovered from the same host individual.

^2^The lineages of two Haemosporida infections were not identified.

## Discussion

Haemosporida are common parasites of suburban birds in North America, yet little is known about their potential interactions with WNV. Our data demonstrate a negative association between the presence of WNV antibodies and avian Haemosporida infection among urban birds of Chicago. However, this negative association was context-dependent, varying with respect to haemosporidian taxonomy and host age. The presence of WNV antibodies was associated with a lower probability of infection with avian *Plasmodium* taxa, but not *Haemoproteus*. Moreover, the inverse association between WNV serostatus and *Plasmodium* infection status was present mainly in adult birds. In contrast to the WNV serostatus effect, birds that had an active WNV infection were equally likely to have a *Plasmodium* infection as birds that did not have a WNV infection. These data suggest that WNV and *Plasmodium* parasites do co-occur and potentially interact within hosts.

Several non-mutually exclusive mechanisms might account for the negative association between the presence of WNV antibodies and the probability of infection with avian *Plasmodium*. First, confounding ecological factors may result in patterns consistent with real interactions between pathogens, even though these interactions do not actually occur within hosts [46]. Shared hosts and vectors predict a passive positive association between the pathogens. However, differing temporal patterns of WNV-seropositive and *Plasmodium*-infected hosts (perhaps related to environmental variables like temperature or vernal recrudescence of previously acquired *Plasmodium* infections [14]) might produce an apparent negative association between the pathogens. We show that *Plasmodium* infections do appear earlier within a transmission season than WNV antibodies among adult hosts in Chicago, IL. However, analyses focused on a period when WNV seropositive and *Plasmodium* infected hosts overlap temporally showed a negative association between the presence of WNV antibodies and *Plasmodium*. This suggests that asynchronous infection dynamics do not solely drive an inverse relationship between WNV serostatus and *Plasmodium* infection status.

Second, WNV and *Plasmodium* may compete directly within a host. Direct competition for host nutrients or cell types could reduce co-occurrence between the pathogens within a host. WNV is known to cause anemia in some birds [47], and thus may reduce the amount red blood cells available to *Plasmodium* parasites. The availability of red blood cells may influence the invasion success of *Plasmodium* parasites, parasitemia, or the persistence of parasites within the bloodstream. For instance, anemia-inducing helminths lower the parasitemia of microparasites that require red blood cells in rodents [4]. However, direct competition would be expected to occur in both juvenile and adult hosts similarly. The apparent restriction of the WNV serostatus effect to adult hosts may suggest this mechanism is less likely.

Third, WNV and *Plasmodium* parasites may interact indirectly, mediated through the host immune system. For instance, a pathogen may “prime” a host’s immune system to respond to a secondary pathogen and thus influence the potential for co-infection [3]. While direct crossover immunity would not be expected between WNV and *Plasmodium* given the biological differences between the pathogens, suppression of one infection by the other has been reported for concurrent infections of *Plasmodium* and other viruses [12]. These effects may be mediated by the Th1 and Th2 polarization of mammalian [48] and avian immune systems [49]. While immune responses to both pathogens are varied, viruses and intracellular microparasites like *Plasmodium* typically activate a Th1 response associated with cell-mediated immunity. Since both WNV and *Plasmodium* elicit the same general cytokine response, the immune response toward one pathogen may also counteract infections by the other [3]. However, *Haemoproteus* may elicit a similar general cytokine response as *Plasmodium.* The lack of a similar relationship between *Haemoproteus* infection and WNV serostatus makes this mechanism somewhat dubious.

Alternatively, co-infection with WNV and *Plasmodium* may reduce host survival, producing an apparent negative association between these pathogens among hosts. Both *Plasmodium* and WNV can produce broad pathological changes in infected avian hosts and fitness consequences for hosts have been documented for each pathogen independently [14, 19, 50, 51]. Co-infections may induce an additive effect on mortality probabilities, either by disrupting important physiological processes, or making death by other extrinsic factors (ie. predation) more likely [52]. Given broad differences in the physiology and causes of mortality between juvenile and adult birds, mechanisms mediated by host physiology could produce different outcomes of pathogen association across age class. Moreover, different patterns of virulence across Haemosporida genera and lineages [11, 14] can lead to different physiological consequences of a WNV co-infection for host individuals.

Identifying the mechanisms that drive an inverse association between WNV serostatus and *Plasmodium* infection status may provide integrated perspectives on host health and demography because alternate mechanisms may impact host survival and disease transmission dynamics differently [3]. For instance, if co-infections elevate the risk of host mortality, *Plasmodium* may have played a role in declines of North American bird populations following the introduction of WNV [24]. Interactions with *Plasmodium* could also impact WNV transmission. If a *Plasmodium* infection primes the immune system against WNV, *Plasmodium* transmission might lower the average host competence for WNV and reduce the potential for WNV transmission. Alternatively, existing *Plasmodium* infections could prolong or intensify a WNV infection, increasing the transmission potential or force of infection exerted by the host. Additionally, if co-infection is associated with increased host mortality, WNV transmission could be impacted by a reduction in recovered hosts that act as sinks in the transmission cycle [53]. Ultimately, controlled experimental infection studies are necessary to understand WNV-*Plasmodium* interactions, and test mechanisms that may produce the inverse association between WNV serostatus and *Plasmodium* infection status among avian hosts. Such studies could shed more light on the implications of potential interactions between these pathogens among wild birds.

## Conclusions

Our results indicate a negative association between West Nile virus serostatus and *Plasmodium* parasites among adult avian hosts within a suburban hotspot of WNV transmission. The correlational nature of the data makes it difficult to identify the mechanism driving this effect. Nevertheless, these results highlight the potential for direct or indirect interactions between these common avian pathogens. Such interactions may have important consequences on host physiology and fitness that may ultimately impact host populations. Further study involving experimental infections are necessary to clarify the mechanisms driving the negative association between WNV and avian *Plasmodium* observed here. Identifying these mechanisms represents a fundamental step toward understanding the potential influence that ubiquitous Haemosporida infections may have on the transmission of a zoonotic pathogen such as WNV.

## Electronic supplementary material

Additional file 1: **This supporting file contains a data summary table, a phylogeny of haemosporidian parasite lineages based on the cytochrome b gene, and full AICc summary tables presenting the results of the analyses presented here.** (DOCX 85 KB)

## Abbreviations

AIC: Aikake information crieteria
AICc: “corrected” Aikake information crieteria
CDC: Centers for disease control and prevention
GLMM: Generalized linear mixed model
WNV: West Nile virus.
