# Supplementary material for: An inverse association between West Nile virus serostatus and avian malaria infection status
Source: Parasit Vectors. 2014 Sep 1;7:415. doi: 10.1186/1756-3305-7-415 (PMC4262112; doi:10.1186/1756-3305-7-415)
Supplement: Supplementary file 1 — Additional file 1: This supporting file contains a data summary table, a phylogeny of haemosporidian parasite lineages based on the cytochrome b gene, and full AICc summary tables presenting the results of the analyses presented here. (DOCX 85 KB) [file 13071_2014_1597_MOESM1_ESM.docx]

**Section I. Supplementary Tables**

Table S1. Table summarizing the dataset.

| Species | N | N (AHY/HY)^1^ | | *Plasmodium*  Prevalence | *Haemoproteus*  Prevalence | WNV  Seroprevalence |
| --- | --- | --- | --- | --- | --- | --- |
| American goldfinch | 144 | 131 | 13 | 0.03 | 0.15 | 0.04 |
| American robin | 425 | 189 | 236 | 0.51 | 0.05 | 0.03 |
| brown-headed cowbird | 19 | 14 | 5 | 0.47 | 0.05 | 0.05 |
| chipping sparrow | 11 | 9 | 2 | 0.09 | .36 | 0.09 |
| common grackle | 50 | 34 | 16 | 0.20 | 0.32 | 0.06 |
| Eurasian starling | 62 | 29 | 33 | 0.18 | 0 | 0.03 |
| gray catbird | 150 | 95 | 55 | 0.17 | 0.16 | 0.07 |
| house finch | 75 | 27 | 48 | 0.20 | 0 | 0.16 |
| house sparrow | 517 | 289 | 228 | 0.17 | 0 | 0.03 |
| mourning dove | 26 | 15 | 11 | 0 | 0.62 | 0.35 |
| northern cardinal | 115 | 70 | 45 | 0.39 | 0.17 | 0.35 |
| red-winged blackbird | 51 | 49 | 2 | 0.14 | 0.16 | 0.10 |
| song sparrow | 69 | 41 | 28 | 0.28 | 0.01 | 0.01 |
| Community Totals | 1714 | 992 | 722 | 0.26 | 0.08 | 0.07 |

^1^After-hatch-year (AHY) and hatch-year (HY) birds

**Section II. Model Selection**

Table S2. AICc table for models predicting Haemosporida infection across all age classes.

| Models | K | AICc | Δ AICc | AICc *w* |
| --- | --- | --- | --- | --- |
| mon+yr+age+wnv+wnv:age+(1\|Species) | 9 | 2056.5 | 0.0 | 0.97 |
| mon+yr+age+wnv+(1\|Species) | 8 | 2064.0 | 7.5 | 0.02 |
| yr+age+wnv+wnv:age+(1\|Species) | 6 | 2069.8 | 13.3 | 0.00 |
| mon+yr+age+(1\|Species) | 7 | 2071.5 | 15.0 | 0.00 |
| mon+yr+wnv+(1\|Species) | 7 | 2072.9 | 16.3 | 0.00 |
| yr+age+wnv+(1\|Species) | 5 | 2077.6 | 21.1 | 0.00 |
| mon+yr+(1\|Species) | 6 | 2077.8 | 21.3 | 0.00 |
| yr+wnv+(1\|Species) | 4 | 2081.8 | 25.3 | 0.00 |
| yr+age+(1\|Species) | 4 | 2083.8 | 27.3 | 0.00 |
| yr+(1\|Species) | 3 | 2086.5 | 30.0 | 0.00 |
| mon+age+wnv+wnv:age+(1\|Species) | 8 | 2088.9 | 32.4 | 0.00 |
| mon+age+wnv+(1\|Species) | 7 | 2098.2 | 41.7 | 0.00 |
| age+wnv+wnv:age+(1\|Species) | 5 | 2100.2 | 43.7 | 0.00 |
| mon+wnv+(1\|Species) | 6 | 2109.3 | 52.8 | 0.00 |
| mon+age+(1\|Species) | 6 | 2109.4 | 52.9 | 0.00 |
| age+wnv+(1\|Species) | 4 | 2109.9 | 53.4 | 0.00 |
| wnv+(1\|Species) | 3 | 2115.9 | 59.4 | 0.00 |
| mon+(1\|Species) | 5 | 2117.2 | 60.7 | 0.00 |
| age+(1\|Species) | 3 | 2119.6 | 63.1 | 0.00 |
| 1+(1\|Species) | 2 | 2123.7 | 67.2 | 0.00 |

Abbreviations: mon= month of capture (4 levels: May/June, July, August, September/October), yr = year of capture (2 levels:2006, 2007), age (2 levels: hatch year juvenile, after hatch-year adult), wnv = WNV serostatus (seropositive, seronegative). Species (13 groups) was a random effect in all models tested. K = # of model parameters, AICc *w*= AICc weight

Table S3. AICc table for models predicting *Plasmodium* infection across all age classes.

| Models | K | AICc | Δ AICc | AICc *w* |
| --- | --- | --- | --- | --- |
| mon+yr+age+wnv+wnv:age+(1\|Species) | 9 | 1704.7 | 0.0 | 0.99 |
| mon+yr+age+wnv+(1\|Species) | 8 | 1713.9 | 9.1 | 0.01 |
| mon+yr+wnv+(1\|Species) | 7 | 1721.9 | 17.2 | 0.00 |
| mon+yr+age+(1\|Species) | 7 | 1725.1 | 20.4 | 0.00 |
| yr+age+wnv+wnv:age+(1\|Species) | 6 | 1727.3 | 22.5 | 0.00 |
| mon+yr+(1\|Species) | 6 | 1730.4 | 25.7 | 0.00 |
| mon+age+wnv+wnv:age+(1\|Species) | 8 | 1731.1 | 26.4 | 0.00 |
| yr+age+wnv+(1\|Species) | 5 | 1737.1 | 32.4 | 0.00 |
| yr+wnv+(1\|Species) | 4 | 1739.3 | 34.6 | 0.00 |
| mon+age+wnv+(1\|Species) | 7 | 1742.2 | 37.5 | 0.00 |
| yr+age+(1\|Species) | 4 | 1745.4 | 40.6 | 0.00 |
| yr+(1\|Species) | 3 | 1746.4 | 41.6 | 0.00 |
| age+wnv+wnv:age+(1\|Species) | 5 | 1748.9 | 44.2 | 0.00 |
| mon+wnv+(1\|Species) | 6 | 1752.4 | 47.6 | 0.00 |
| mon+age+(1\|Species) | 6 | 1757.7 | 53.0 | 0.00 |
| age+wnv+(1\|Species) | 4 | 1760.6 | 55.9 | 0.00 |
| mon+(1\|Species) | 5 | 1764.4 | 59.6 | 0.00 |
| wnv+(1\|Species) | 3 | 1764.5 | 59.7 | 0.00 |
| age+(1\|Species) | 3 | 1772.7 | 68.0 | 0.00 |
| 1+(1\|Species) | 2 | 1774.8 | 70.1 | 0.00 |

Abbreviations: mon= month of capture (4 levels: May/June, July, August, September/October), yr = year of capture (2 levels:2006, 2007), age (2 levels: hatch year juvenile, after hatch-year adult), wnv = WNV serostatus (seropositive, seronegative). Species (13 groups) was a random effect in all models tested. K = # of model parameters, AICc *w*= AICc weight

Table S4. AICc table for models predicting *Haemoproteus* infection across all age classes.

| Models | K | AICc | Δ AICc | AICc *w* |
| --- | --- | --- | --- | --- |
| mon+(1\|Species) | 5 | 742.3 | 0.0 | 0.32 |
| mon+yr+(1\|Species) | 6 | 744.1 | 1.8 | 0.13 |
| mon+age+(1\|Species) | 6 | 744.2 | 1.9 | 0.12 |
| mon+wnv+(1\|Species) | 6 | 744.3 | 2.0 | 0.12 |
| mon+yr+age+(1\|Species) | 7 | 746.1 | 3.8 | 0.05 |
| mon+yr+wnv+(1\|Species) | 7 | 746.1 | 3.8 | 0.05 |
| mon+age+wnv+(1\|Species) | 7 | 746.2 | 3.9 | 0.05 |
| 1+(1\|Species) | 2 | 746.7 | 4.4 | 0.04 |
| mon+age+wnv+wnv:age+(1\|Species) | 8 | 747.6 | 5.3 | 0.02 |
| mon+yr+age+wnv+(1\|Species) | 8 | 748.0 | 5.6 | 0.02 |
| age+(1\|Species) | 3 | 748.2 | 5.9 | 0.02 |
| yr+(1\|Species) | 3 | 748.5 | 6.1 | 0.01 |
| wnv+(1\|Species) | 3 | 748.7 | 6.4 | 0.01 |
| mon+yr+age+wnv+wnv:age+(1\|Species) | 9 | 749.4 | 7.1 | 0.01 |
| yr+age+(1\|Species) | 4 | 749.9 | 7.6 | 0.01 |
| age+wnv+(1\|Species) | 4 | 750.2 | 7.9 | 0.01 |
| yr+wnv+(1\|Species) | 4 | 750.5 | 8.2 | 0.01 |
| age+wnv+wnv:age+(1\|Species) | 5 | 751.6 | 9.2 | 0.00 |
| yr+age+wnv+(1\|Species) | 5 | 751.9 | 9.6 | 0.00 |
| yr+age+wnv+wnv:age+(1\|Species) | 6 | 753.3 | 11.0 | 0.00 |

Abbreviations: mon= month of capture (4 levels: May/June, July, August, September/October), yr = year of capture (2 levels:2006, 2007), age (2 levels: hatch year juvenile, after hatch-year adult), wnv = WNV serostatus (seropositive, seronegative). Species (13 groups) was a random effect in all models tested. K = # of model parameters, AICc *w*= AICc weight

Table S5. AICc table for models predicting *Plasmodium cathemerium* infection across all age classes.

| Models | K | AICc | Δ AICc | AICc *w* |
| --- | --- | --- | --- | --- |
| mon+yr+age+wnv+(1\|Species) | 8 | 823.4 | 0.0 | 0.59 |
| mon+yr+age+wnv+wnv:age+(1\|Species) | 9 | 825.2 | 1.8 | 0.24 |
| mon+yr+age+(1\|Species) | 7 | 827.4 | 4.0 | 0.08 |
| yr+age+wnv+(1\|Species) | 5 | 827.8 | 4.4 | 0.07 |
| yr+age+wnv+wnv:age+(1\|Species) | 6 | 829.5 | 6.2 | 0.03 |
| yr+age+(1\|Species) | 4 | 831.9 | 8.5 | 0.01 |
| mon+yr+wnv+(1\|Species) | 7 | 836.0 | 12.7 | 0.00 |
| mon+age+wnv+(1\|Species) | 7 | 841.3 | 18.0 | 0.00 |
| age+wnv+(1\|Species) | 4 | 842.1 | 18.7 | 0.00 |
| yr+wnv+(1\|Species) | 4 | 842.7 | 19.3 | 0.00 |
| mon+age+wnv+wnv:age+(1\|Species) | 8 | 842.9 | 19.5 | 0.00 |
| mon+yr+(1\|Species) | 6 | 843.1 | 19.8 | 0.00 |
| age+wnv+wnv:age+(1\|Species) | 5 | 843.6 | 20.3 | 0.00 |
| mon+age+(1\|Species) | 6 | 849.1 | 25.8 | 0.00 |
| yr+(1\|Species) | 3 | 849.2 | 25.8 | 0.00 |
| age+(1\|Species) | 3 | 849.5 | 26.2 | 0.00 |
| mon+wnv+(1\|Species) | 6 | 850.9 | 27.6 | 0.00 |
| wnv+(1\|Species) | 3 | 854.0 | 30.6 | 0.00 |
| mon+(1\|Species) | 5 | 862.6 | 39.2 | 0.00 |
| 1+(1\|Species) | 2 | 864.5 | 41.2 | 0.00 |

Abbreviations: mon= month of capture (4 levels: May/June, July, August, September/October), yr = year of capture (2 levels:2006, 2007), age (2 levels: hatch year juvenile, after hatch-year adult), wnv = WNV serostatus (seropositive, seronegative). Species (13 groups) was a random effect in all models tested. K = # of model parameters, AICc *w*= AICc weight

Table S6. AICc table for models predicting *Plasmodium elongatum* infection across all age classes.

| Models | K | AICc | Δ AICc | AICc *w* |
| --- | --- | --- | --- | --- |
| yr+age+wnv+(1\|Species) | 5 | 586.2 | 0.0 | 0.40 |
| yr+age+wnv+wnv:age+(1\|Species) | 6 | 587.0 | 0.8 | 0.27 |
| yr+age+(1\|Species) | 4 | 587.6 | 1.4 | 0.20 |
| mon+yr+age+wnv+(1\|Species) | 8 | 590.7 | 4.5 | 0.04 |
| mon+yr+age+wnv+wnv:age+(1\|Species) | 9 | 591.5 | 5.3 | 0.03 |
| yr+wnv+(1\|Species) | 4 | 591.9 | 5.7 | 0.02 |
| mon+yr+age+(1\|Species) | 7 | 592.0 | 5.8 | 0.02 |
| age+wnv+(1\|Species) | 4 | 594.2 | 8.0 | 0.01 |
| age+wnv+wnv:age+(1\|Species) | 5 | 594.5 | 8.3 | 0.01 |
| yr+(1\|Species) | 3 | 595.1 | 8.9 | 0.00 |
| mon+yr+wnv+(1\|Species) | 7 | 595.5 | 9.3 | 0.00 |
| wnv+(1\|Species) | 3 | 597.8 | 11.5 | 0.00 |
| age+(1\|Species) | 3 | 598.1 | 11.9 | 0.00 |
| mon+yr+(1\|Species) | 6 | 598.9 | 12.6 | 0.00 |
| mon+age+wnv+(1\|Species) | 7 | 599.9 | 13.7 | 0.00 |
| mon+age+wnv+wnv:age+(1\|Species) | 8 | 600.2 | 14.0 | 0.00 |
| mon+wnv+(1\|Species) | 6 | 602.8 | 16.5 | 0.00 |
| mon+age+(1\|Species) | 6 | 603.7 | 17.5 | 0.00 |
| 1+(1\|Species) | 2 | 603.8 | 17.6 | 0.00 |
| mon+(1\|Species) | 5 | 609.0 | 22.8 | 0.00 |

Abbreviations: mon= month of capture (4 levels: May/June, July, August, September/October), yr = year of capture (2 levels:2006, 2007), age (2 levels: hatch year juvenile, after hatch-year adult), wnv = WNV serostatus (seropositive, seronegative). Species (13 groups) was a random effect in all models tested. K = # of model parameters, AICc *w*= AICc weight

Table S7. AICc table for models predicting Haemosporida infection across all adult birds.

| Models | K | AICc | Δ AICc | AICc *w* |
| --- | --- | --- | --- | --- |
| yr+wnv+(1\|Species) | 4 | 1138.9 | 0.0 | 0.69 |
| mon+yr+wnv+(1\|Species) | 7 | 1140.9 | 2.1 | 0.24 |
| yr+(1\|Species) | 3 | 1144.4 | 5.5 | 0.04 |
| mon+yr+(1\|Species) | 6 | 1145.5 | 6.7 | 0.02 |
| wnv+(1\|Species) | 3 | 1157.7 | 18.9 | 0.00 |
| mon+wnv+(1\|Species) | 6 | 1160.0 | 21.1 | 0.00 |
| 1+(1\|Species) | 2 | 1168.2 | 29.4 | 0.00 |
| mon+(1\|Species) | 5 | 1169.2 | 30.4 | 0.00 |

Table S8. AICc table for models predicting Haemosporida infection across all juvenile birds.

| Models | K | AICc | Δ AICc | AICc *w* |
| --- | --- | --- | --- | --- |
| mon+yr+(1\|Species) | 6 | 862.7 | 0.0 | 0.73 |
| mon+yr+wnv+(1\|Species) | 7 | 864.7 | 2.0 | 0.27 |
| mon+(1\|Species) | 5 | 876.5 | 13.8 | 0.00 |
| mon+wnv+(1\|Species) | 6 | 878.5 | 15.8 | 0.00 |
| yr+(1\|Species) | 3 | 892.1 | 29.4 | 0.00 |
| yr+wnv+(1\|Species) | 4 | 893.9 | 31.2 | 0.00 |
| 1+(1\|Species) | 2 | 904.7 | 41.9 | 0.00 |
| wnv+(1\|Species) | 3 | 906.4 | 43.7 | 0.00 |

Abbreviations: mon= month of capture (4 levels: May/June, July, August, September/October), yr = year of capture (2 levels:2006, 2007), wnv = WNV serostatus (seropositive, seronegative). Species (13 groups) was a random effect in all models tested. K = # of model parameters, AICc *w*= AICc weight

Table S9. AICc table for models predicting *Plasmodium* infection across all adult (after hatch-year) birds.

| Models | K | AICc | Δ AICc | AICc *w* |
| --- | --- | --- | --- | --- |
| yr+wnv+(1\|Species) | 4 | 871.4 | 0.0 | 0.64 |
| mon+yr+wnv+(1\|Species) | 7 | 872.6 | 1.2 | 0.35 |
| yr+(1\|Species) | 3 | 880.6 | 9.2 | 0.01 |
| mon+yr+(1\|Species) | 6 | 882.4 | 10.9 | 0.00 |
| wnv+(1\|Species) | 3 | 883.9 | 12.5 | 0.00 |
| mon+wnv+(1\|Species) | 6 | 886.7 | 15.3 | 0.00 |
| 1+(1\|Species) | 2 | 897.4 | 26.0 | 0.00 |
| mon+(1\|Species) | 5 | 900.9 | 29.5 | 0.00 |

Table S10. AICc table for models predicting *Plasmodium* infection across all juvenile (hatch-year) birds.

| Models | K | AICc | Δ AICc | AICc *w* |
| --- | --- | --- | --- | --- |
| mon+yr+(1\|Species) | 6 | 785.5 | 0.0 | 0.72 |
| mon+yr+wnv+(1\|Species) | 7 | 787.4 | 1.9 | 0.28 |
| mon+(1\|Species) | 5 | 799.9 | 14.4 | 0.00 |
| mon+wnv+(1\|Species) | 6 | 801.8 | 16.3 | 0.00 |
| yr+(1\|Species) | 3 | 804.2 | 18.7 | 0.00 |
| yr+wnv+(1\|Species) | 4 | 806.2 | 20.7 | 0.00 |
| 1+(1\|Species) | 2 | 815.7 | 30.2 | 0.00 |
| wnv+(1\|Species) | 3 | 817.7 | 32.2 | 0.00 |

Abbreviations: mon= month of capture (4 levels: May/June, July, August, September/October), yr = year of capture (2 levels:2006, 2007), wnv = WNV serostatus (seropositive, seronegative). Species (13 groups) was a random effect in all models tested. K = # of model parameters, AICc *w*= AICc weight

Table S11. AICc table for models predicting *Plasmodium* infection across adult birds caught during the latter half of the transmission season (July-October).

| Models | K | AICc | Δ AICc | AICc *w* |
| --- | --- | --- | --- | --- |
| yr+wnv+(1\|Species) | 4 | 381.4 | 0.0 | 0.52 |
| mon+yr+wnv+(1\|Species) | 6 | 381.7 | 0.3 | 0.44 |
| wnv+(1\|Species) | 3 | 388.5 | 7.1 | 0.01 |
| mon+wnv+(1\|Species) | 5 | 389.5 | 8.2 | 0.01 |
| mon+yr+(1\|Species) | 5 | 389.6 | 8.2 | 0.01 |
| yr+(1\|Species) | 3 | 389.8 | 8.4 | 0.01 |
| 1+(1\|Species) | 2 | 400.3 | 18.9 | 0.00 |
| mon+(1\|Species) | 4 | 401.3 | 20.0 | 0.00 |

Abbreviations: mon= month of capture (4 levels: May/June, July, August, September/October), yr = year of capture (2 levels:2006, 2007), wnv = WNV serostatus (seropositive, seronegative). Species (13 groups) was a random effect in all models tested. K = # of model parameters, AICc *w*= AICc weight

**Section III. Haemosporida Phylogeny**

Figure S1. Phylogenetic analysis of all Haemosporida cytochrome *b* lineages by maximum likelihood assuming a GTR+γ substitution model of evolution (γ parameter = 0.3257). Numbers above or below branches indicate bootstrap support (%) estimated from 1,000 resamplings of the sequence data. Branches with ≤ 50% bootstrap support are collapsed. The tree is mid-point rooted. Branch lengths are not drawn to scale. There are 48 unique parasite haplotypes presented here, some of which have been grouped into independent lineages (see Methods for criteria). Lineages denoted by PA are *Haemoproteus,* while those denoted by PL are *Plasmodium*. This tree was primarily used to assign lineages to one of these genera. *Plasmodium cathemerium* and *Plasmodium elongatum* are the lineages CHI03PL and CHI06PL, respectively. Both selection of the substitution model by BIC score and phylogenetic reconstruction were conducted in MEGA5 (Tamura *et al.* 2011).

References

Tamura K, Peterson D, Peterson N, Stecher G, Nei M, Kumar S: MEGA5: Molecular evolutionary genetics analysis using maximum likelihood, evolutionary distance, and maximum parsimony methods. *Molecular Biology and Evolution* 2011, **28**:2731–2739.
